# Transportation, childcare, lodging, and meals: Key for participant engagement and inclusion of historically underrepresented populations in the healthy brain and child development birth cohort

**DOI:** 10.1017/cts.2024.4

**Published:** 2024-02-12

**Authors:** Aleksandra E. Zgierska, Tatum Gramly, Nicholas Prestayko, Danielle Symons Downs, Traci M. Murray, Lea G. Yerby, Brittany Howell, Barbara Stahlman, Jennifer Cruz, Arjola Agolli, Holly Horan, Florence Hilliard, Julie M. Croff, the HEALthy Brain and Child Development (HBCD) Consortium

**Affiliations:** 1 Departments of Family and Community Medicine, Anesthesiology and Perioperative Medicine, and Public Health Sciences, Pennsylvania State University College of Medicine, Hershey, PA, USA; 2 Department of Family and Community Medicine, Pennsylvania State University College of Medicine, Hershey, PA, USA; 3 Department of Kinesiology and College of Medicine, Department of Obstetrics and Gynecology, Pennsylvania State University College of Health and Human Development, University Park, PA, USA; 4 National Institutes of Health, National Institute on Drug Abuse, Bethesda, MD, USA; 5 Department of Community Medicine and Population Health, The University of Alabama College of Community Health Sciences, Tuscaloosa, AL, USA; 6 Department of Human Development and Family Science, Virginia Tech, Fralin Biomedical Research Institute, Roanoke, VA, USA; 7 Department of Obstetrics and Gynecology, Global and Rural Women’s Health Program, Heersink School of Medicine, The University of Alabama at Birmingham, Birmingham, AL, USA; 8 University of Wisconsin-Madison Waisman Center, Madison, WI, USA; 9 Oklahoma State University Center for Health Sciences, Tulsa, OK, USA

**Keywords:** Childcare, lodging, meal, recruitment, retention, transportation

## Abstract

**Introduction:**

Participant recruitment and retention (R&R) are well-documented challenges in longitudinal studies, especially those involving populations historically underrepresented in research and vulnerable groups (e.g., pregnant people or young children and their families), as is the focus of the HEALthy Brain and Child Development (HBCD) birth cohort study. Subpar access to transportation, overnight lodging, childcare, or meals can compromise R&R; yet, guidance on how to overcome these “logistical barriers” is sparse. This study’s goal was to learn about the HBCD sites’ plans and develop best practice recommendations for the HBCD consortium for addressing these logistical barriers.

**Methods:**

The HBCD’s workgroups developed a survey asking the HBCD sites about their plans for supporting research-related transportation, lodging, childcare, and meals, and about the presence of institutional policies to guide their approach. Descriptive statistics described the quantitative survey data. Qualitative survey responses were brief, not warranting formal qualitative analysis; their content was summarized.

**Results:**

Twenty-eight respondents, representing unique recruitment locations across the U.S., completed the survey. The results indicated substantial heterogeneity across the respondents in their approach toward supporting research-related transportation, lodging, childcare, and meals. Three respondents were aware of institutional policies guiding research-related transportation (10.7%) or childcare (10.7%).

**Conclusions:**

This study highlighted heterogeneity in approaches and scarcity of institutional policies regarding research-related transportation, lodging, childcare, and meals, underscoring the need for guidance in this area to ensure equitable support of participant R&R across different settings and populations, so that participants are representative of the larger community, and increase research result validity and generalizability.

## Introduction

Participant recruitment and retention (R&R) represent well-known challenges in human subjects research (HSR) [1], especially with studies involving young children, long-term follow-up, burdensome assessments, and/or interventions or procedures with some level of risk. Inadequate R&R can threaten the integrity of research and its findings [2]. Lack of access to affordable, reliable transportation, childcare, overnight lodging, or meals are some of the common logistical issues surrounding study activities and can pose major barriers to research participation. The differences in needs and perceived barriers versus motivators can be subtle yet important between different communities. Researchers can reduce burden and foster positive experiences for participants by proactively addressing potential barriers in advance of a study, and by practicing procedures to systematically assess and meet evolving, often unanticipated needs of the study participants.

Transportation-related challenges may make it impossible for some individuals to engage in research requiring in-person visits [3]. Improved access to public transportation, especially taxi or rideshare services, could help boost recruitment [4]. Residential proximity to the research site and reliable public transportation can increase willingness of individuals to participate in research [5]. Unfortunately, 45% of Americans do not have access to public transport [6]. For rural residents, limited public transportation and concerns about parking are major barriers to participation [7]. Even if participants have transportation, they may have to cover the related costs upfront, and then receive reimbursement later, posing challenges, especially for participants with lower socioeconomic status (SES). These issues can be further compounded if car seats are required for child transportation.

Lack of available or affordable childcare is another major barrier. Securing childcare has been reported as “extremely difficult” by participants [8–11], and identified as one of the most important facilitators for research participation among pregnant and breastfeeding persons [12]. Participants with less-flexible work schedules may require childcare to enable their participation [13]. Yet, many families are unable to afford childcare, and/or the availability of childcare can be limited, especially in rural areas [14]. Although some research groups can provide onsite childcare, many sites do not have access to a pool of volunteers (e.g., undergraduate or graduate students) or a child-friendly space.

Lack of affordable lodging near the research site can be an insurmountable barrier to research participation when overnight accommodations are needed, e.g., due to participant extended travel time to the research site, the duration or timing of the study visits, or participant safety concerns. As with other barriers, this can especially impact those with low SES and rural residents. Freidman *et al*. (2015) noted that perceived recruitment barriers, motivators, and strategies were contextually similar between rural and urban sites; however, the perception of the importance of certain factors varied, with rural participants paying more attention to the study-related time commitment and benefits to the entire family [15]; strategies increasing the perception that study participation is “worth their time” and emphasizing family aspects may help boost research engagement in rural areas.

Participant access to food, whether snacks or larger meals, increases participant satisfaction with research and the likelihood they will complete the study [1,16,17]. Providing food and drinks, especially during longer study visits, ensures participants are not distracted by hunger or thirst. This acknowledgment of participants’ biological needs is particularly important for studies involving pregnant, lactating, or child participants. Shared meal times by offering a less formal social environment and a positive atmosphere can be an opportunity to enhance trust and rapport between research team and participants [18].

The intersectionality of participant needs and logistical barriers to research participation has been well-documented, and addressing these challenges can enhance representative population sampling, which is critical for robust conclusions to be drawn from any research. Persistently low enrollment rates are common in research, causing extended enrollment periods and delays in research completion [19]. Even with a representative sample enrolled at baseline, external validity can be challenged in longitudinal research by attrition, which is anticipated to be higher among participants from disadvantaged social backgrounds, minority groups, or who are pregnant, younger, low-income, less educated, in unstable marital partnerships, have mental illness, or use substances [9,16]. Groups historically underrepresented in research include racial, ethnic, sexual, and gender and other minority groups; geographically isolated groups (e.g., rural populations or residential racial segregation); vulnerable populations, including the elderly, pregnant people, children, individuals with disabilities, limited English proficiency [20], and fewer economic resources. These groups have been impacted by negative historical factors and social determinants of health known to increase health inequities and reduce research participation [14]. Previous studies have found that family and work obligations and stressful life events are more frequently experienced by marginalized and underrepresented groups, limiting their capacity to engage in research despite their desire to do so, and requiring research/researcher flexibility and support [1,21]. In addition, our understanding of how to best meet the needs of gender and sexual minority groups and effectively engage them in research is still evolving. Employing participant-centered, culturally sensitive practices that foster trust between researchers and participants, and anticipating and overcoming logistical burdens can improve research engagement, particularly among populations historically underrepresented in research [9,16,22,23].

This is of particular concern for the NIH Helping to End Addiction Long-term® (HEAL) HEALthy Brain and Child Development (HBCD) study [15], which focuses on young children whose parents may need to bring their other children to the study visits, especially since the HBCD’s assessments can be long and its brain magnetic resonance imaging (MRI) is best acquired during a child’s evening/nighttime sleep when childcare support volunteers are harder to recruit.

The HEAL HBCD long-term birth cohort study, focused on inclusion of vulnerable populations, may be particularly affected by these considerations [24]. The HBCD study plans to engage a diverse population of 7,500 parent (or guardians or other caregivers) - child dyads, starting with pregnant people who are representative of the US population, to better understand child development from pregnancy through early childhood. The design of the HBCD study combines longitudinal assessments of brain (using the MRI and electroencephalogram), cognitive and behavioral development, biospecimens, contextualized by in-depth characterization of the pre- and post-natal environments through the first decade of child’s life. The study protocol includes several study visits (both remote and in-person) that span from several hours to multiple days, bringing to light questions about best practices for equitable research engagement of participants from diverse populations. Given its national scope, it is critical for the HBCD study to understand factors that may negatively impact R&R, so that it can positively and effectively engage participants, including those from historically underrepresented and marginalized groups.

With this in mind, and with a dearth of evidence to inform practical solutions for improving R&R of participants in longitudinal research, we developed a study-specific survey and surveyed the research sites participating in the HBCD consortium prior to study launch to learn about their local strategies and plans for meeting participants’ needs related to transportation, lodging, childcare, and meals. This manuscript presents this survey’s findings on the current landscape of these support strategies, followed by recommendations for overcoming logistical barriers and supporting families and children across the HBCD consortium as the essential first step toward developing equitable and adaptable best practices.

## Materials and methods

### Design

The survey was an outcome of the discussions held with members from the NIH HEAL HBCD study [15] sites and workgroups. Site members discussed how they planned to support participants’ transportation, childcare, lodging, and meal/snack needs during study visits. This project did not meet criteria for HSR and did not require review by the Institutional Review Board.

### Survey design

The survey was developed by investigators from the HBCD study’s Rural and Sovereign Communities Workgroup, with input from the members of the consortium-wide R&R; Diversity, Equity, and Inclusion; Ethics, Legal, and Policy; and Study Navigators Workgroups. This non-validated survey was tailored to the HBCD study needs and administered in the planning phase of HBCD, prior to participant enrollment, and designed to capture the spectrum of planned strategies related to participant transportation, childcare, lodging, and meal/snack support needs, so that the survey-yielded data could serve as a platform for developing guidance for all sites regarding best practices for strategies to address participant needs during research activities. Most questions offered multi-choice responses and options for qualitative comments to describe individual site’s plans not captured by the closed-ended response choices. The final survey (see Appendix 1) included 20 questions, querying sites about their location, strategies and barriers regarding transportation, childcare, lodging, and meals/snacks, and one open-ended question: “*Anything else you would like to share about transportation, lodging, childcare or meal related considerations or issues?*”

### Procedures

A link to the Qualtrics survey (Qualtrics, Version October 2022, Provo, UT) was sent by the consortium’s Administrative Core to all sites’ Principal Investigators and completed between 10/08/2022 through 11/15/2022. The survey collected information identifying the site, but not information about the person who completed the survey on the site’s behalf. Although the HBCD study includes 25 sites, some sites include more than one location, resulting in 28 consortium recruitment locations (i.e., potential respondents).

### Analytical approach

Frequencies of responses were calculated across survey respondents using Microsoft Office 2019 Excel program, with the total sample of completed surveys serving as the denominator for all percentage calculations. Qualitative responses were reviewed by the authors to determine the extent to which they provided additional contextual information and were grouped into general themes. The overall number, length, and type of the qualitative comments did not meet the standards for formal qualitative thematic analytic procedures [25]. The results were categorized by participant need type (e.g., transportation, childcare, etc.).

## Results

All 25 funded institutions, representing 28 recruitment locations completed the survey; therefore, *n* = 28 was used as a denominator to calculate the frequencies of specific responses. Among the survey respondents, 100% answered the multiple-choice survey questions, and 25% (7 respondents) provided responses to the open-ended question.

### Transportation

The sites reported various approaches for supporting participant transportation to and from study appointments. Most respondents (25 [89.3%]) planned to arrange for taxi, transportation service, or rideshare programs (e.g., ZipCar, Uber, Lyft). Sixteen respondents (57.1%) reported no barriers to these services, six (21.4%) did not or could not arrange for such services, and six (21.4%) qualitatively described rideshares as being unreliable in their area, unavailable outside of city limits, or limited by institutional policies.

Paying upfront for transportation, without any cost to the participant, was the dominant approach (24 [85.7%]). In addition, 16 respondents (57.1%) planned to reimburse participants based on the mileage driven to/from the study site, and six (21.4%) planned to reimburse participants after they covered travel-related expenses. Thirteen respondents (46.4%) also planned on supporting transportation for all participants, while 14 respondents considered specific criteria for offering transportation, by participant request (14 [50.0%]), if the participant lives over one hour away (7 [25.0%]), or if the study visit spanned two days (4 [14.3%]). Eleven respondents (39.3%) added qualitative responses about offering rideshares, participant reimbursement through gas cards or paying for onsite parking, or research staff driving participants to the study site or meeting participants at the participant-selected locations.

In the event a participant did not have a required car seat for child transportation, 12 respondents (42.9%) planned to provide one, and 10 (35.7%) planned to make them available through the arranged transportation service. Nine (32.1%) also marked having “other plans,” such as getting car seats from local community organizations or providing study-owned car seats for vehicles used to transport participants (e.g., Uber Medical; institutional fleet). Seven respondents (25.0%) reported not having plans for car seats yet. Eighteen respondents (64.3%) wished to have rear-facing, 17 respondents wished to have forward-facing (60.7%) car seats available, and 14 (50.0%) planned to have booster seats in the future.

Fourteen respondents (50.0%) had plans for research personnel or approved volunteers to travel to meet participants in the community for study-related activities, while 4 respondents (14.3%) planned to do this “in general, but not right now,” and 9 (32.1%) did not plan to do it.

When asked about the liability and personal injury coverage when driving participants or driving to meet participants, 16 respondents (57.1%) did not respond, and nine (32.1%) did not know if their institution provided such coverage.

### Childcare

One respondent (3.6%) did not plan to provide childcare for siblings/children accompanying participants during the visits. Twenty-seven respondents (96.4%) planned to provide childcare, using designated study staff (22 [78.6%]) and/or trained volunteers (21 [75.0%]) onsite, and/or making provisions for “ecological support” (i.e., space for the parent/caregiver to care for their child/children). The majority (16 [57.1%]) planned to offer childcare support whenever requested.

Only three respondents (10.7%) stated their institution has a policy guiding childcare for research participation; 12 respondents (42.9%) answered “No,” and 11 respondents (39.3%) answered “I donʼt know” regarding such policies.

### Lodging

Six respondents (21.4%) did not plan to offer participants overnight lodging. Most (17 [ 60.7%]) planned to offer lodging near the site, with five respondents (17.9%) still working on a specific plan. When asked about their criteria for offering overnight lodging for in-person visits, 11 respondents (39.3%) did not answer this question. The remaining respondents planned to offer lodging if the visit ended late at night (17 [60.7%]), if there were concerns about participant safety when returning home late (13 [46.4%]), if the assessments spanned two consecutive days (9 [32.1%]), or if the participant lived over an hour away (9 [32.1%]). Notably, only one respondent (3.6%) planned to offer lodging to all participants, regardless of circumstance.

### Meals/snacks

The respondents endorsed varied plans for feeding participants and/or parents/caregivers during the study visits. Twenty-six (92.9%) planned to offer shelf-stable snacks/drinks; 18 (64.3%) planned to offer baby foods, including rice cereal; and 15 (53.6%) planned to offer bottles and formula. Fourteen respondents (50.0%) also planned to provide vouchers for participants to purchase food at a local restaurant, with 11 respondents having a list of selected restaurants for participants to choose from, and 3 respondents not having the details established yet. Twenty-two respondents (78.6%) planned to offer snacks/meals to all, at every in-person visit.

### Other

Three respondents commented in response to: “*Anything else you would like to share…?,*” noting the survey helped them identify issues and areas they had not previously considered, and that it would be useful to receive specific guidance and funding/financial assistance to help overcome these types of logistical barriers.

## Discussion

Findings from this cross-sectional survey of members from 28 unique data collection locations (across 25 awarded research sites) of the HBCD consortium conducting a large-scale, long-term birth cohort study across the U.S. [24] highlight critical considerations and plans for addressing logistical barriers related to R&R, including transportation, childcare, lodging, and meals for research participants. Plans for addressing participant needs varied across the locations, despite an otherwise standardized, common study protocol. Responses also emphasized that institutional policies are often inadequate (or missing), thus insufficient for effectively guiding these aspects of HSR that are critical for recruitment and retention, which, in turn, are essential for the validity and generalizability of research findings. Notably, the sites involved in the HBCD study largely comprise experienced research teams from academic institutions with a long history of HSR. Yet, even these teams continue to grapple with logistical barriers, highlighting an urgent need for developing guidance on these issues to ensure equitable support for research participants across diverse study settings and populations.

The limited evidence and recommendations on how to conceptualize these “mundane” aspects of research, combined with sparse or non-existent institutional policies, place a burden on individual research teams to create detailed participant support protocols, while also navigating legal and regulatory concerns. These concerns are particularly relevant to research-related transportation and childcare considerations. Providing safe transportation and childcare requires advanced planning, secure facilities, and an adequate number of sufficiently trained and approved staff or volunteers. Yet, although most institutions enforce child protection training and safety guidelines for campus-based youth programs, many do not carry these policies further or provide guidelines specific to research projects involving children and families. Similar challenges relate to transportation, when an institution may guide their faculty/staff driving in general terms (in personal, institutional fleet, or externally rented vehicles) but not in relation to research participant transportation and relevant legal/financial aspects.

Our survey findings indicated substantial heterogeneity in approaches across the study recruitment locations, and a scarcity of policies and published guidelines related to the “logistical barriers.” Yet, overcoming barriers to research participation involves addressing both the logistical barriers and tangible resources (including transportation, childcare, lodging, and meals) and intangible ones. Ongoing engagement with participants, their families, and communities is critical toward understanding and accommodating their needs, building trust and rapport, and creating a more equitable participant experience. Integrating input from patients (“peers”) and other stakeholders into study protocols can positively change these historically harmful power dynamics; patient stakeholders become research partners, acknowledged as subject matter experts of their own needs, and work together with researchers to develop effective solutions addressing logistical barriers [26,27]. This is key in building an atmosphere, in which participants feel valued, heard, and able to honestly and timely voice emergent needs. Involving “peers” (e.g., recovery peer specialists in a substance use-related research) can boost research engagement among hard-to-reach, vulnerable populations [28]. Hiring research personnel who speak the same language as the potential study participants (i.e., “bilingual staff”), are trained in cultural competency and conditions under the investigation, and avoid stigmatizing language can also improve recruitment rates, more than focusing on hiring ethnically matched study personnel [29]. It is important to understand that different study sites will (and should have) different engagement plans, which reflect participant needs at each site. Even within a site, plans to increase equity and retain diverse participants should be multifaceted, offering a range of supports to meet varying needs of individuals, communities, and research teams. Working with stakeholder partners can help research teams identify both local barriers and solutions optimally suited for their local contexts to support participation in research of diverse groups.

The success of the HBCD study and similar HSR with in-person assessments will hinge on consistent, honest, long-term participation from diverse families who volunteer to commit to a major investment of their time and effort. It is the responsibility of research teams, as those with the funding resources, to support equitable research participation in order to conduct impactful, robust investigations. Therefore, we propose a set of recommendations for the HBCD consortium regarding transportation, childcare, lodging, and meals to support research participants based on the existing literature and experience of researchers involved in the HBCD study (Table 1). Although we focused on these specific areas, they do not exhaust a list of potential logistical barriers to research that participants may face (e.g., language or mobility-related barriers). It is important for research teams to consider specific needs of the study population during the planning phase of each project and adequately budget for overcoming the identified barriers. Future surveys of the HBCD sites about their approach to addressing logistical barriers, along with the evaluation of actual R&R outcomes across the sites, will provide data to better discern if our recommendations and site-applied specific strategies can increase engagement of participants across diverse populations, including those historically underrepresented in research. What follows are the HBCD study team recommendations regarding the provision of transportation, childcare, lodging, and meals as a means of equitably supporting participant study engagement.

**Table 1.** Recommendations for minimizing the impact of transportation, childcare, lodging, and meals as “logistical barriers” to participant research engagement

Level	Recommendations
1. Funding agencies	a) Consider requiring research proposals to outline plans (e.g., within the recruitment and retention section of NIH grant applications) and associated estimated expenses for overcoming logistical barriers, with budgets and strategies tailored to the needs of each study and local circumstances; such plans can help ensure consistency and equitability of supports across participants and study sites, and identification of study-specific “logistical barriers” should be part of the study planning and budgeting processes.
	b) Consider requiring involvement in research of appropriate stakeholders as advisors and partners to increase the likelihood of tailoring the research protocols to account for the needs of the study population.
2. Research institutions	a) Have policies in place regarding research-related transportation, childcare, lodging, and meals; such policies are critical for safe, equitable research conduct and access.
	b) For research-related transportation, these policies should outline personal injury and liability coverage details to ensure that research personnel or approved volunteers do not (unknowingly) expose themselves and the study to the risks of personal injury/liability-related costs, for example, when driving research participants, especially when participant transportation requires providing and/or installing car seats for children.
	c) Regarding childcare, these policies should specify who can offer childcare support, and the way this support can be provided, following appropriate laws/regulations; the liability coverage should be clarified upfront for the study personnel or volunteers who assist with childcare. Institutions can further support child safety by offering or facilitating “certificate programsʼʼ specifically designed for individuals interacting with children in research settings.
	d) Consider establishing institutional-level arrangements / agreements with service providers (e.g., taxi, rideshare, car rental companies, hotels, restaurants, childcare centers, or providers) to reduce burden otherwise placed on individual research teams, and promote participant/staff safety, and institutional-level regulatory compliance.
	e) Support a diverse menu of reimbursement options to research participants (such as gift cards from different vendors, checks, cash, or debit cards, e.g., ClinCard) and offering upfront (rather than retrospective) reimbursements to reduce the risk of unintentional discrimination against and deterrence of certain populations from research engagement; for example, the need for providing a social security number or detailed personal information, proficiency with/access to online transactions, or the need to pay first then be reimbursed can negatively affect participation by groups historically underrepresented in research.
	f) Consider providing research space tailored to child, family, and community needs (e.g., child/family-friendly research room; additional room appropriate for child play; private space for breastfeeding) in order to promote equitable research engagement.
3. Research teams	a) Ensure the approach for addressing logistical barriers is consistent with the institutional/local and funding agency policies and regulations, and IRB-approved.
	b) Offer an upfront coverage (from the research funds) of expenses related to logistical barriers, rather than requesting participants to pay out-of-pocket first, before providing a retrospective reimbursement; not all participants/families can afford upfront expenses.
	c) Offer assistance with transportation, childcare, lodging, or snack/meal to all (prospective and current) participants, especially during in-person study visits. Training of the research staff in universally-offering these services and conveying related messages in a non-judgmental way is vital; scripting of such communication and trauma-informed training can be useful. This universal approach may increase the study cost in the short term, but the benefit to the rigor of the scientific work merits this investment; these expenses, covered from research funds, should be carefully monitored and inform future research considerations. The universal assistance approach can help “normalize” the use of support services, and reduce stigmatization and stereotyping of certain groups of participants, helping counter the negative perspective many community members from historically underrepresented groups may have of researchers and research studies. In addition, many individuals do not feel comfortable asking for help and would rather miss a study visit than ask directly for assistance.
	d) When hiring research personnel, studies should convey upfront if there is a need for staff’s availability during nontraditional work hours in order to meet participant scheduling needs, and consider equitable compensation for the variability and unpredictability of the study personnel schedules that the study might require.
	e) Hiring a study navigator (e.g., recovery peer support specialist or other support professional with lived experience relevant to the study population) as a research team member could be beneficial for addressing participant needs and increasing recruitment and retention as some participants may be more open and comfortable with “peers” than the “traditional” research staff.
4. Transportation	a) Researchers should consider transportation barriers and strive to provide transportation or alternative methods of study participation (e.g., virtual) to those unable to complete in-person visits. For example, providing gas-vouchers (or other forms of financial support to offset fuel cost) upfront, prior to the study visit, can promote visit attendance, supporting participants from lower-resourced communities.
	b) Study protocols should describe permitted versus unpermitted modes/types of transportation (e.g., taxi, rideshare services, etc.), and the conditions under which research personnel or approved volunteers can drive participants to/from the research site or drive to meet participants at participant-preferred locations, following institutional policies and IRB-approved plans.
5. Childcare	a) Researchers should consider potential childcare-related barriers and their impact on participant research engagement for both in-person and remote study visits, and make childcare accessible, so that everyone can participate in research; this includes arrangements for offering upfront financial support for at-home childcare.
	b) Individuals involved in childcare assistance should complete child abuse-related training to reduce the risk of child abuse/neglect, and ensure child safety and compliance with child abuse reporting mandates, and receive “clearance” based on their criminal background check prior to being involved in childcare activities.
	c) Childcare services delivered by providers hired or contracted specifically for this task should be institutionally approved, with proper agreements in place. If offering “ecological support” to the parents/caregivers at the study site, the protocol should outline who (e.g., study personnel, volunteers), where (i.e., on site versus not) and how (e.g., what is allowed versus not, e.g., the need for at least two people to be always present? who can change diapers?) will assist parents/caregivers with childcare, and what training requirements these individuals need to satisfy.
6. Overnight lodging	a) Lodging should be offered to all study participants, especially when research procedures are lengthy or conducted later in the evening.
	b) Selection of overnight lodging options should be aligned with the needs of participants and their accompanying families (e.g., proximity to research site; room(s) large enough to support a family unit) and the institutional requirements regarding approved costs for lodging in the area; although institutions may have preferred vendors who meet the approved cost criteria, alternative lodging options should be considered if the “standard” approved ones are less convenient for participants and/or their families. Planning these arrangements in advance can reduce the burden of adding a new vendor.
7. Snacks/meals	a) Snacks/meals to support research participants and their families may be necessary, especially during longer visits. Cost of snacks/meals may be regulated both by institutional and federal policies, and should be considered when developing study-specific plans.
	b) A healthy snack should be offered during all in-person visits; recording participant preferences and offering a “preferred snack” may be particularly appreciated by participants and their families, especially for those with dietary or allergy considerations. A full meal should be considered for longer visits or visits during traditional meal times (e.g., breakfast, lunch, or dinner).
	c) Children and pregnant or lactating persons may require more frequent or particular snacks/meals than other groups. Being mindful of individual needs is especially important when research participants receive vouchers to eat in nearby restaurants, which may not have appropriate snacks/food; arranging for alternative snack/meal options/locations can help meet participant needs and support research engagement. Engaging a nutritionist or dietician to advise on appropriate healthy foods can be helpful.

## Conclusions

Funding agencies and research institutions can facilitate engagement of diverse participants in HSR by aligning their funding supports and policies to overcome common logistical barriers and support R&R and equity in research participation. Researchers must take a multifaceted approach to R&R to ensure that study activities are appealing, accessible, and conducted within a welcoming, inclusive environment for all participants. The strategies and their impact on R&R should be continually evaluated to inform result validity, generalizability, interpretation, and future approaches. The scientific imperative to ensure that study participants are representative of the larger community is dependent on addressing barriers, which have led to historical underrepresentation of some groups in research.

## Supporting information

Zgierska et al. supplementary material Zgierska et al. supplementary material

## Supplementary material

The supplementary material for this article can be found at https://doi.org/10.1017/cts.2024.4.
