# Supplementary material for: Transportation, childcare, lodging, and meals: Key for participant engagement and inclusion of historically underrepresented populations in the healthy brain and child development birth cohort
Source: J Clin Transl Sci. 2024 Feb 12;8(1):e38. doi: 10.1017/cts.2024.4 (PMC10928703; doi:10.1017/cts.2024.4)
Supplement: Zgierska et al. supplementary material [file S2059866124000049sup001.doc]

**HEALthy Brain and Child Development (HBCD) Study Transportation, Lodging, Childcare, and Meals Survey**

Considerations related to transportation, overnight accommodations, childcare and meals are very important to all HBCD study sites, from urban to rural. Several HBCD Study workgroups have partnered to put together this survey and learn about each site’s plans for supporting participants and overcoming study participation barriers related to transportation, overnight accommodations, childcare or meals. Some of these issues may also involve policy/legal/regulatory considerations, and approval from your institution and/or the Institutional Review Board before implementation. Please note that your site’s plans should be consistent with your institutional policies, which can usually be found on institutional policy or risk management websites.

Please complete this survey by answering the following questions. Each question allows for an optional free-text entry – please share with us your plans, thoughts, concerns. Your comments and suggestions are sincerely appreciated! After we close the survey, all the responses will be compiled and shared across the HBCD Study Consortium.

**QUESTION 1. Which HBCD Study site are you associated with?** _______________

**QUESTION 2. How do you intend to support participant transportation to / from study appointments?** (check all that apply)

- 1. **Arrange for a ride through a free community transportation program** (If selected: Please provide the name and/or more information about this program: _____________)
  2. **Provide public transit vouchers, e.g., bus passes**
  3. **Arrange for a taxi, transportation service, or ride share program**
  4. **Utilize existing institutional transportation system (e.g., university-based shuttle)**
  5. **Employee-research team member will drive participant(s)**
  6. **Volunteer team member will drive participant(s)**
  7. **Reimburse participants for travel expenses based on the mileage**
  8. **Other approaches or additional comments:** ______________________
  9. **We don’t know yet**
  10. **We are not planning to support participant transportation to/from study appointments** (if selected: Please describe why: _______________)

If response 2c was selected:

**QUESTION 3.** Please provide more detail (check all that apply):

- - 1. Participant will pay then be reimbursed by the study
    2. The study will pay for it (no cost to participant at any point)

If response 2e was selected:

**QUESTION 4. Please provide more detail (check all that apply):**

- - - - 1. In an institutional fleet vehicle, i.e., the vehicle is owned by the institution (if selected: Please provide more information: Does your institution require background checks or special registration or certification for an employee to use a fleet vehicle? _______________)
      1. In a personal vehicle (if selected: Please provide more information: Does your institution specify a level or type of insurance an employee must have to be reimbursed for use of a personal vehicle for university business? ___________)

If response 2f was selected:

**QUESTION 5. Please provide more detail (check all that apply):**

- - - - 1. In an institutional fleet vehicle, i.e., the vehicle is owned by the institution (if selected: Please provide more information: Does your institution require background checks or special registration or certification for a volunteer to use a fleet vehicle? _______________)
    1. In a personal vehicle (if selected: Please provide more information: Does your institution specify a level or type of insurance a volunteer must have to be reimbursed for use of a personal vehicle for university business? __________)

**QUESTION 6. Are there barriers to ride share or other transportation programs at your site (e.g., ZipCar, Uber, Lyft)?** (check all that apply)

- - - 1. **No barriers**
      2. **Ride share or rental are prohibited by our institutional policies** (if selected: Provide the names of prohibited ride share programs and reason of prohibition: _______________)
      3. **Other situation** (if selected: Please tell us more: _____________________________)
      4. **We have not yet begun the process to contract with a ride share company**

**QUESTION 7. If you are planning to support participant transportation, when do you intend to do it?** (check all that apply)

- 1. **When participant lives > 1 hour drive time from the study appointment site**
  2. **When the study assessments need to be split over two consecutive days**
  3. **When participant requests**
  4. **We plan to offer transportation support to all participants, regardless of the above**
  5. **Other** (if selected: Please describe: ______________________)
  6. **We don’t know yet**

**QUESTION 8. What is your site’s plan in the event a participant does not have a car seat for child transportation (when car seat is required according to the child’s age)?** (check all that apply)

- 1. **We will provide a car seat to participant when needed at no cost**
  2. **Car seats will be available through the contracted or arranged transportation**
  3. **We plan to have car seats available, but the details have not been finalized yet**
  4. **Other plan** (if selected: Please share details: _______________________)
  5. **We do not plan to provide a carseat**

If responses 8 a, b, c or d were selected:

**QUESTION 9: Please provide: What types of car seats do you plan to have available to offer to the participants?** (check all that apply)

1. Rear-facing
2. Forward-facing
3. Booster seat
4. Don’t know yet

**QUESTION 10. Does your site have plans for research team personnel or approved volunteers to travel to where participants live or a place of their choosing in the community to complete activities related to recruitment, enrollment or some of the study assessments?**

**a. No**

**b. Not yet, but plan to do it**

**c. Yes** (if selected, please share details: ______________________)

If responses 10 a or b were selected:

**QUESTION 11. At your site, who is covered by liability and personal injury coverage for individual drivers of research participants or when driving to complete research activities?** (check all that apply)

- 1. Employees when driving a university-owned/operated vehicle
  2. Authorized volunteers when driving a university-owned/operated vehicle
  3. Employees when driving a personal vehicle for research purposes
  4. Authorized volunteers when driving a personal vehicle for research purposes
  5. Contracted drivers as part of the contract
  6. Other coverage (if selected: Please share details: _________________)
  7. Our institution does not provide liability coverage
  8. I don’t know whether liability coverage is provided

**QUESTION 12. What type of overnight lodging are you planning to offer to participants near the study appointment site?** (check all that apply)

**a. Hotel/motel**

**b. Airbnb**

**c. VRBO**

**d. Other** (if selected: Please share details: __________________________)

**e. We don’t intend to offer lodging**

**f. We don’t yet have a plan for lodging**

If responses 12 a, b, c or d were selected:

**QUESTION 13. If you are planning to support participant overnight lodging, when do you intend to do it?** (check all that apply)

- 1. When participant lives > 1 hour drive time from the study appointment site
  2. When the study assessments need to be split over two consecutive days
  3. When the study appointment ends late in the evening/night, and there are concerns about safety when returning home late
  4. When participant requests
  5. We plan to offer lodging support to all participants, regardless of the above
  6. Other (if selected: Please share details: ________________________)
  7. We don’t know yet

**QUESTION 14. What are your site’s plans for child care of siblings/children accompanying participants during the study assessment visits?** (check all that apply)

1. **Contract with a childcare group to provide child care on site**
2. **Contract with a childcare group to provide child care at the participant’s home**
3. **Ecological support (e.g., provide space when the caregiver can take care of their other child/children)**
4. **Designated study staff to support families on site**
5. **Designated trained volunteer (e.g., an undergraduate student) to support families on site**
6. **Reimburse families for the cost of childcare, which they secure themselves**
7. **We plan to support child care, but do not details finalized yet**
8. **We do not plan to support child care** (if selected: Please explain why: ________________)

If responses 14 a, b, c, d, e, f or g were selected:

**QUESTION 15: If you are planning to support child care, when do you intend to do it?** (check all that apply)

- 1. When participant lives > 1 hour drive time from the study appointment site
  2. When a given study assessment takes a long time
  3. When the study appointment ends late in the evening/night
  4. When participant requests
  5. We plan to offer childcare support to all, regardless of the above
  6. Other (if selected: Please describe: _________________________)
  7. We don’t know yet

**QUESTION 16. Does your institution have a policy guiding child care for research participation?**

1. **No**
2. **I don’t know**
3. **Yes** (if selected: Please specify: __________________________)

**QUESTION 17. What are your site’s plans for feeding participants and/or caregivers during the study assessments?** (check all that apply)

1. **Bottles and formula**
2. **Rice cereal and baby foods**
3. **Shelf-stable snacks / drinks**
4. **Frozen meals**
5. **Order food from a local restaurant (within established per diem costs)**
6. **Provide a voucher for participants to purchase food at the local restaurant (within established per diem costs)**
7. **Ask participants to bring their own food**
8. **We plan to offer snacks/meals but do not have details finalized yet**
9. **We do not plan to provide snacks/meals for participants**

If responses 17 a, b, c, d, e, f, g or h were selected:

**QUESTION 18: If you are planning to support snacks/meals, when do you intend to do it?** (check all that apply)

- 1. When participant lives > 1 hour drive time from the study appointment site
  2. When a given study assessment takes a long time
  3. When the study appointment ends late in the evening/night
  4. When participant requests
  5. We plan to offer snacks/meals to all, regardless of the above
  6. Other (please describe) ______________________
  7. We don’t know yet

If responses 17 e or f were selected:

**QUESTION 19: If you plan to provide meals through a local restaurant, how do you intend to do it?**

a. Pre-specified single restaurant

b. Pre-specified set of restaurants for participant to choose from

c. We do not have detailed plans yet

**Question 20. Anything else you would like to share about transportation, lodging, childcare or meal related considerations or issues..?** ______________________
